# Toward Understanding the Role of Aryl Hydrocarbon Receptor in the Immune System: Current Progress and Future Trends

**DOI:** 10.1155/2014/520763

**Published:** 2014-01-06

**Authors:** Hamza Hanieh

**Affiliations:** Biological Sciences Department, King Faisal University, P.O. Box 380, Hofouf 31982, Saudi Arabia

## Abstract

The immune system is regulated by distinct signaling pathways that control the development and function of the immune cells. Accumulating evidence suggest that ligation of aryl hydrocarbon receptor (Ahr), an environmentally responsive transcription factor, results in multiple cross talks that are capable of modulating these pathways and their downstream responsive genes. Most of the immune cells respond to such modulation, and many inflammatory response-related genes contain multiple xenobiotic-responsive elements (XREs) boxes upstream. Active research efforts have investigated the physiological role of Ahr in inflammation and autoimmunity using different animal models. Recently formed paradigm has shown that activation of Ahr by 2,3,7,8-tetrachlorodibenzo-p-dioxin (TCDD) or 3,3′-diindolylmethane (DIM) prompts the differentiation of CD4^+^Foxp3^+^ regulatory T cells (Tregs) and inhibits T helper (Th)-17 suggesting that Ahr is an innovative therapeutic strategy for autoimmune inflammation. These promising findings generate a basis for future clinical practices in humans. This review addresses the current knowledge on the role of Ahr in different immune cell compartments, with a particular focus on inflammation and autoimmunity.

## 1. Introduction

The aryl hydrocarbon receptor (Ahr) is a transcription factor belonging to the basic helix-loop-helix-Per-Arnt-Sim (bHLH-PAS) superfamily of proteins, which is ubiquitously expressed in vertebrate cells [1, 2]. For many years, Ahr has been described to mediate the mechanisms that undertake the environmental toxicity and immunotoxicity [3]. Nevertheless, later progress has expanded Ahr functions much beyond to include aspects of the circadian rhythm, reproduction, redox potential, autoimmunity, and various cellular processes [4–10]. These multiple physiological functions of Ahr have been confirmed using different animal models including Ahr-null mice, hypomorphs, and more recently cell-specific conditional deletions of Ahr.

In immunological aspects, Ahr is abundant in most immune cells, if not all, albeit at different levels, reviewed in [11]. It mediates the proliferation, differentiation, and cytokines secretion of adaptive and innate immune cell compartments, in particular T helper (Th)-17, regulatory T cells (Tregs), and dendritic cells (DCs) that play profound roles in autoimmunity [12]. Furthermore, many inflammatory response-related genes have potential xenobiotic-responsive elements (XREs) embedded in upstream sequences. It is likely that these polymorphisms can be transcriptionally regulated by Ahr, and, therefore, modulate various autoimmune diseases.

Accumulating evidence has clearly linked Ahr to several murine autoimmune models including experimental autoimmune encephalomyelitis (EAE) [13, 14], ulcerative colitis (UC) [15, 16], collagen-induced arthritis (CIA) [7], and Sjögren's syndrome [17]. However, the underlying mechanisms are largely anonymous. This review provides current state of the art on the role of Ahr in the differentiation and functions of different immune cell compartments, focusing in particular on inflammation and autoimmunity. In addition, the paper discusses the proposed mechanistic details that undertake the actions of Ahr.

## 2. Ahr: Background and Biochemistry

**Figure 1:**
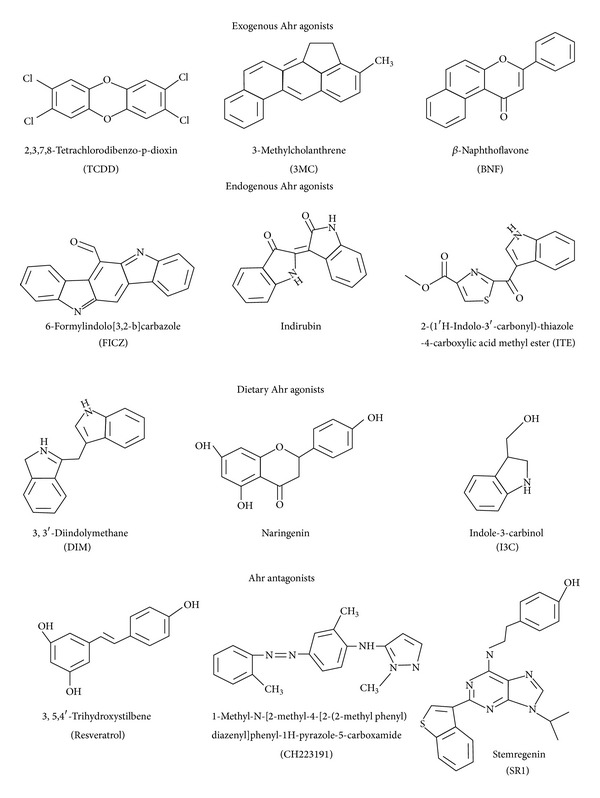
2-dimensional structure of selected Ahr agonists and antagonists.

The Ahr is a heterodimeric ligand-dependent protein activated by a structurally diverse variety of ligands, which fit minimum requirements of aromaticity and hydrophobicity (Figure 1). The endogenous Ahr ligands are a long-listed group of chemicals synthesized in the organism, including indigoids, equilenin, and metabolites of arachidonic acid, heme, and tryptophan, which are highlighted in recent reviews [2, 11, 18, 19]. The Ahr is activated vigorously by the tryptophan photoproducts 6-formylindolo-[3,2-b]-carbazole (FICZ) and 6,12-diformylindolo-[3,2-b]-carbazole (dFICZ) that are induced by UV in the skin. More recently, kynurenine (Kyn), the tryptophan metabolite induced by indoleamine 2,3-dioxygenase (IDO), is considered as an Ahr agonist [20]. Further tryptophan metabolite, the 2-(1′H-indole-3′-carbonyl)-thiazole-4-carboxylic acid methyl ester (ITE), is reported to exert Ahr activation in lungs fibroblasts [21] and myofibroblast [22]. The essential role of tryptophan in Ahr activation suggests that more derived tryptophan metabolites may be able to activate Ahr and arelikely to be characterized in the future. Furthermore, the naturally occurring Ahr ligands are predominantly found in plants such as certain flavonoids and glucosinolates [9, 23, 24].

Since the discovery over 3 decades [25], Ahr has been intensively studied for its physiological significance using man-made ligands. The best-characterized exogenous ligands of Ahr, which attract the attention of many groups, are a wide variety of ubiquitous polycyclic aromatic hydrocarbons (PAHs), halogenated aromatic hydrocarbons (HAHs), and polychlorinated biphenyls (PCB) compounds, reviewed in [18]. The xenobiotic 2,3,7,8-tetrachlorodibenzo-p-dioxin (TCDD), as named by toxicologist, is the most potent Ahr ligand that binds with high affinity and stability. Its chlorine residues are considered advantageous for Ahr research since they prevent metabolic breakdown, which sustain prolonged Ahr signaling, and today TCDD is the Ahr agonist of choice. Conversely, the long half-life of TCDD is a hindrance, since the prolonged activation of Ahr deregulates responsive genes and the consequent outcomes. Additionally, exposure to TCDD exerts diversity of toxic and biological effects, including tumor promotion and immuno-, hepato-, cardio-, and dermal toxicity, reviewed in [26].

Residing in cytosol, Ahr complexes with the chaperons prostaglandin E synthase 3 (P23), heat shock protein (Hsp)-90, and Ahr-interacting protein (AIP). This dimerization keeps Ahr inactive and prevents proteolysis. Activation allows Ahr to dissociate these chaperons and expose the N-terminal, which facilitates the translocation of Ahr/ligand complex into the nucleus. In the classical pathway, Ahr dimerizes with another bHLH-PAS member the Ahr nuclear translocator (Arnt) or hypoxia-inducible factor (HIF)-1*β*, reviewed in [27, 28]. The Ahr/Arnt heterodimer binds to the small conserved promoter elements XREs, also termed dioxin-responsive elements (DREs), which recruits cofactors (p300, cAMP, CREB, and RIP140) to accelerate expression of the responsive genes [29]. The Ahr mediates toxicity via upregulation of gene expression of the xenobiotic-metabolizing enzymes such as *Cyp1A1*, *Cyp1A2*, *Cyp1B1*, *Gst1A1*, *Ugt1a6,* and *Aldh3a1*, reviewed in [1, 30]. A second conserved Ahr promoter element, Ahr-II, has been identified, which recognizes Ahr with additional cofactor(s) only [31]. However, the paucity of studies has investigated the physiological consequences of Ahr binding to the Ahr-II.

The signaling pathway of Ahr is conserved and can work independently or in interaction with other pathways. Proof that Ahr cross talks with different pathways is already established, which seemingly adds more complexity to the mechanism of Ahr action. For instance, we and others have shown that Ahr interacts with signal transducers and activators of transcription (STAT) 1 [32], nuclear factor (NF)-*κ*B subunits [33], transacting transcription factor (Sp1) [34], estrogen receptor (ER), and kinases such as src [35]. Hundreds of genes incorporated in a wide range of physiological responses have the core-pentanucleotide sequence (5′-TNGCGTG-3′) of the XREs [29]. Therefore, it is to be suggested that the alternative pathways specify the Ahr responsive genes and create downstream differences in a condition-specific fashion.

## 3. Ahr in Adaptive Immune Cells

An adaptive immune response is triggered via activation, differentiation, and clonal expansion of the lymphoid lineage cells, T and B lymphocytes. This response is an overall outcome of a multitude of environmental and genetic factors. Since a long time ago, many immunotoxicological studies have clearly demonstrated that Ahr impairs immune responses to a variety of pathogens such as the influenza virus [36], herpesviruses [17], and *Streptococcus pneumoniae* [37]. The Ahr exerts its immunosuppression via drastic changes in thymic involution, thymocyte and T cell apoptosis, shifts in immune cell subsets, and splenic atrophy, reviewed in [26, 38].

Depending on the signal received, the T cells proceed to differentiate along transcription factor-specific pathways that give distinct T cell subsets. These lineage-specific transcription factors include T-bet for Th1, GATA3 for Th2, ROR*γ*t for Th17, and Foxp3 for Tregs. Interestingly, applying the promoter sequence analysis software, that is, TF search, shows that these transcription factors have potential XREs boxes in upstream sequences, albeit at different frequencies, assuming that activation of Ahr may directly/indirectly modulate the commitment of these T cell subsets. Nevertheless, the functional importance of these XREs in lineage commitment remains unclear.

### 3.1. Th1 and Th2

The expression of Ahr is not detected in mice Th1 and Th2 cells during differentiation [39]. However, application of Ahr agonists shifts the Th1/Th2 balance towards Th1 dominance by GATA3 inhibition, which ameliorates allergy [39] and allergic asthma [40] in mice models. Taken together, it may be expected that Ahr is implicated in modulation rather than in initiation of the normal differentiation of Th1/Th2 cells. The upstream sequence of* Gata3* contains a high frequency of potential XREs boxes, while Ahr activation suppresses the Th2 differentiation. Therefore, more investigation should provide important mechanistic explanations.

Treating mice with TCDD ameliorates allergic sensitization by the inhibition of interleukin (IL)-4 [41]. Cytokines analysis during allergic sensitization with DNP-Ascaris extracts shows higher IL-5 production and IgE titre in Ahr-null mutant (B6Ahr^D1/D1F^) mice [39]. Relevant results using an allergic lung inflammation model show higher IL-5 production in Ahr^−/−^ mice [42]. The stimulation of splenocytes from Ahr^D1/D1G^ mice augments interferon (IFN)-*γ* and IL-12 production [43]. On the other hand, TCDD treatment suppresses the Th1 effector function by reducing IFN-*γ* production [13, 14]. This suppression may be attributed to the enhanced IL-10 that interrupts JAK-STAT pathway. In agreement, the inhibition of Ahr by resveratrol increases Th1 and Th2 cells [39], which is supported by our findings in the CIA model using Ahr^−/−^ mice [7].

### 3.2. Th17 and Tregs

The Ahr plays pivotal roles in the differentiation and functions of the CD4^+^ effector cells Th17 and Tregs, the hallmark of autoimmunity (see Box 1). Data from several groups reveal that Ahr-deficient mice have a defective differentiation of these T cell subsets and that Ahr is highly expressed in both human [44] and mice Th17 [13, 32, 44] cells. Consistently, activation of Ahr by certain agonists enhances Th17 expansion in both species [32, 44]. Mechanistic investigations unravel that the interaction of Ahr with STAT5 down-regulates STAT5 phosphorylation and hence reduces IL-2 production, resulting in an induction of Th17 cells [32, 45].

Also, it is suggested that, under Th17-polarizing conditions, STAT3 and Ahr upregulate the expression of Aiolos [46]. Using Aiolos-deficient mice, the authors demonstrate that Aiolos silences the *Il2* locus, promoting the Th17 differentiation *in vitro* and *in vivo*. More recently, our data show that activation of Ahr by FICZ enhances the Th17 differentiation via upregulating microRNA (miR)-212 that targets B-cell lymphoma (Bcl)-6, a negative regulator of Th17, and that miR-132/212-deficient mice have an impaired differentiation of this T cell subset [47].

The IL-17- and IL-22-producing Th17 cells contribute to the host defense against extracellular pathogens, and they are implicated in the pathogenesis of autoimmune disorders. The interaction between Ahr and IL-22 was first reported when Veldohoen and colleagues [44] demonstrated that Th17 from Ahr^−/−^ mice did not produce IL-22 and that FICZ treatment in the wild type increased the production of IL-17 and IL-22 in these cells, which worsened EAE. In related studies in human memory T cells, it was shown that FICZ or TCDD increased the IL-22 production but not that of IL-17 [48, 49]. In contrast to the observations in mice, when the effects of FICZ on the differentiation of IL-17- and IL-22-producing cells were studied, the data showed that FICZ promoted IL-22-producing cells but inhibited the generation of Th17 [48].

Under Th17-polarizing conditions, Ahr interacts with JAK-STAT pathway to exert its modulatory effects on the production of IL-17 and IL-22 by Th17 cells [32]. Consistently, retroviral transductions of *Ahr* alone or combined with *RORc2* under different skewing conditions, except for Th17 cells, are insufficient to induce the production of IL-17 and IL-22 [38, 44]. Interesting data indicate that nitric oxide (NO) suppresses the differentiation and functions of polarized human and murine Th17 cells, and deletion of inducible NO synthase (iNOS) in mice (Nos2^−/−^) results in more severe EAE manifested by an increased production of IL-17A [50]. The authors suggest that NO also inhibits the expression of Ahr in Th17 cells concomitant with the inhibition of IL-22, IL-23r, and CYP1A1. In this regard, liver x receptor (LXR) suppresses the differentiation of Th17 cells and the expression of IL-17 [51, 52]. Mechanistically, LXR-induced Srebp-1 inhibits the IL-17 transcription through a cross talk with Ahr that inhibits binding of Ahr to the *Il17* promoter [52].

It is now clearly evident that activation of Ahr enhances the differentiation of Tregs. For instance, activation of Ahr by TCDD induces CD4^+^Foxp3^+^ Tregs *in vitro* and *in vivo* [13, 14, 32]. The role of Ahr in promoting the differentiation of Tregs is further confirmed using Kyn [53] and VAF347 [54]. Recent studies suggest that the naturally occurring Ahr ligands such as diindolylmethane (DIM) [55] and naringenin [9] stimulate the induction of CD4^+^Foxp3^+^ Tregs.

The Tregs from Ahr-deficient mice show lower levels of IL-10 [56, 57]. This may support that the Ahr-deficient mice have elevated levels of IL-12 and IFN-*γ*, the cytokines inhibited by IL-10 [58, 59]. Along the same line, Ahr physically interacts with c-maf to control the transcriptional activity of the *Il10* promoter in Tregs [57]. Interestingly, Ahr/c-maf heterodimer likely plays a role in the differentiation of Th17 cells via regulating IL-22 expression [60]. These observations may support that Ahr is essential, but works differentially, in the differentiation and functions of Th17 and Tregs.

New Tregs that express Foxp3 and produce IL-10 have been identified in mice, so-called Tr1. It is suggested that Ahr enhances the generation of these cells and mediates the production of IL-10 and IL-21 via interaction with c-maf, resulting in amelioration of EAE [56]. Relevant findings show that the probiotic *Bifidobacterium breve* induces IL-10-producing Tr1 cells that express Ahr, c-maf, and IL-21 and that DCs treated with this probiotic are capable of inducing Tr1 cells in mice [40]. In humans, activation of Ahr in the presence of TGF-*β*1 induces CD4^+^Foxp3^+^ Tregs, which suppresses responder T cells through different mechanisms including the ectonucleoside triphosphate diphosphohydrolase CD39 [59]. The latter mechanism is also conceivable since CD39 deficiency is associated with an exacerbated autoimmune inflammation in human and murine models. For a recent review, see [61].

### 3.3. Th22

The Th22 cells, with the CCR6^+^CCR4^+^CCR10^+^ phenotype, are a distinct T cell subset with specific genes expression, in which Ahr is the key transcription factor. These cells produce IL-22, but not IL-17, which is upregulated in patients with autoimmune diseases including systemic lupus erythematosus (SLE) [63], multiple sclerosis (MS) [64], rheumatoid arthritis (RA) [65], and Sjögren's syndrome [66]. Therefore, Th22 cells may be an additional player in the scenario of autoimmunity.

Direct exposure to TCDD favors the emergence of IL-22-secreting CD4^+^ cells in humans [49]. Consistently, VAF347 promotes the differentiation of naïve CD4^+^ into IL-22-secreting cells [67]. The data from the same study suggest that VAF347 prompts the differentiation of human monocytes into DCs capable of directing the differentiation of naïve CD4^+^ cells to secrete IL-22 but concomitantly inhibits IL-17. Therefore, Ahr selectively enhances IL-22 in Th22 cells. However, it remains unclear what the mechanistic explanation of this selectivity might be, though the promoter regions of *Il17* and *Il22* contain XREs boxes, and whether this selectivity could be of therapeutic benefits.

### 3.4. CD8^+^ T Cells

Although the role of Ahr in CD4^+^ cells has been intensively studied, more studies are required to address the physiological significance of Ahr in CD8^+^ cells (CTLs). Several groups have shown that TCDD suppresses the proliferation and differentiation of influenza virus-specific CTLs, reviewed in [58]. However, mechanistic studies are limited, which makes it difficult to interpret. Notably, the suppressive effect of TCDD on DCs during influenza infection is also reported [36], and the early CD8^+^ functions during viral infection are CD4^+^ independent. Therefore, it may be presumed that TCDD suppresses CTLs functions during viral infection indirectly by suppressing the DCs.

The modulatory effects of Ahr on the differentiation and functions of CTLs have been studied in allograft aspects. Activation of Ahr is associated with suppression of the allospecific CTLs responses through a mechanism that is Ahr dependent. However, for TCDD to suppress alloresponse, treatment must occur before activation of the CTLs [68], suggesting that Ahr may be necessary for the initial stages of CTLs activation.

Sharing some commonalities with CD4^+^ cells, TCDD-activated Ahr enhances CD25 expression on CD8^+^ cells, which induces a CTL phenotype termed CD8^+^ Tregs that are capable of suppressing responder T cells and proinflammatory cytokines [69]. Deregulation of these cells has been recently linked to certain animal models of autoimmunity such as EAE [70], UC [71], and CIA [72]. However, the significance of Ahr in these cells in relevance to autoimmunity is awaiting further investigation.

### 3.5. B Cells

Little is known about the role of Ahr in the differentiation of B cells. It has been shown that Ahr deficiency in B cells is associated with more immature cells, while TCDD treatment reduces the mature cells [73]. Activation of Ahr impairs humoral immunity at many endpoints [74] and increases susceptibility to infections [36, 75]. For example, TCDD mediates the reactivation induction of EBV, a ubiquitous herpesvirus that infects >90% of the world's population, by transactivating BZLF1, an Ahr responsive gene, which consequently can be a risk factor for Sjögren's syndrome [17].

The TCDD treatment in mice decreases the number of IgM-secreting plasma cells and delays the differentiation of B cells by inhibition of the activator protein (AP)-1 and the activation of the transcription factor Bach2 [76]. In lines, TCDD suppresses IgE production in mice models while inhibiting IgM in transformed mice cell line, reviewed in [77]. The suppressive effects of TCDD on Ig expression in mice may be mediated, at least in part, by the repression of the 3′Igh regulatory region (3′*IghRR*) [78]. Although the number of published reports studying human B cells is limited, the available data show that the sensitivity of human and mice B cells to suppression by TCDD is comparable; see [77]. In contrast to the data in mice, TCDD activates the 3′*IghRR*, suggesting species differences in the mode of action of TCDD. Interestingly, hs1,2, the enhancer of 3′*IghRR,* has XREs in the invariant sequence [78], and it has been correlated with many autoimmune diseases in humans [79–81], suggesting that TCDD may influence autoimmune inflammation through regulation of the hs1,2.

Activation of Ahr by ITE suppresses the differentiation of B cells into Ig-secreting plasma cells and the production of IgM, IgE, and IgG1 [82]. In an effort to provide mechanistic details, they found that the activation of Ahr suppresses the mRNA expression of secreted-type Ig and plasma cell-specific genes such as *γ*1GLT and *ɛ*GLT that are necessary for class switching. However, some questions are still unanswered. For example, what are the active interplays of Ahr with other signaling pathways in B cells?

## 4. Ahr in Innate Immune Cells

Stimulation of the myeloid lineage cells including granulocytes, macrophages (MΦ), DCs, and natural killer (NK) cells not only forms the innate immunity as a first line of defense, but also shapes the subsequent adaptive responses. A number of outstanding reviews have addressed the modulatory effect of Ahr in innate immune responses [11, 30, 83]. Herein, the review presents an overview of recent reports that investigated the potential role of Ahr in the differentiation and function of innate immune cells as well as the suggested mechanistic illustrations.

Fundamental genes that are relevant to the innate immune response have different frequencies of XREs in the promoter regions, including, all toll-like receptors (TLRs), complement genes, IL-6, TNF-*α*, and IL-1r [62]. Furthermore, Ahr expression is detected in the cells of the innate immune system, such as DCs, MΦ, and NK cells; see [11]. It is congruent, therefore, that activation of Ahr in these cells modulates different features of the innate immune response.

### 4.1. Neutrophils

Neutrophils are the most abundant leukocytes that mediate acute inflammatory response to numerous infections. Therefore, the role of Ahr in these cells has been studied synergistically in response to challenge. For example, TCDD treatment increases the recruitment of neutrophils in the lungs following influenza virus infection in mice, which worsens inflammation [84, 85]. Interestingly, the genes of granulocyte-macrophage colony-stimulating factor (GM-CSF), granulocytes (G)-CSF, and the neutrophil chemoattractant keratinocyte chemoattractant (KC) contain no XREs boxes, suggesting an indirect mode of action by TCDD that may include nonimmune cells. Supporting this notion, it is reported recently that elevated iNOS levels and neutrophil numbers in the influenza virus-infected lung result from Ahr-dependent signaling in endothelial and respiratory epithelial cells, respectively [84].

In contrast, Veiga-Parga et al. [86] found that neutrophils and proinflammatory cytokines were less in the corneas of herpesvirus simplex-infected mice with TCDD treatment. In response to *Streptococcus pneumoniae* infection, neutrophils numbers were not increased in the TCDD-treated animals, and, notably, there was a trend of decreased number of phagocytes recovery in the lung lavage [37]. Furthermore, TCDD treatment before pleuritis initiation reduced the number of neutrophils during inflammation [87]. It is likely that the differences in TCDD administration time and/or dose contributed to the above-mentioned discrepancies.

### 4.2. DCs and MΦ

Available data clearly indicate that DCs play prominent roles in autoimmune inflammation, which can be modulated by activation of Ahr. For example, TCDD promotes Tregs development indirectly by inducing IDO in DCs, which enhances Foxp3 expression [88]. Along the same line, ligation of Ahr by ITE induces tolerogenic dendritic cells (DCs) that are capable of enhancing the differentiation of Tregs [89]. However, the underlying mechanisms of these modulations remain to be defined.

To perform their function in bridging innate and adaptive immunities, immature DCs undergo substantial changes upon TLRs ligation, including the expression of costimulatory molecules and secretion of cytokines. It is quite evident from earlier and recent *in vitro* studies that Ahr ligation enhances DCs maturation and T cell stimulation capacity. These effects are characterized by the elevated expression of costimulatory molecules such as MHCII, CD40, CD54, CD80, CD86, and CD274 [90, 91].

The Fms-like tyrosine kinase 3 ligand (Flt3L) is used to induce the immature steady-state DCs that reside in peripheral immune tissues, whereas GM-CSF is used to generate the so-called inflammatory DCs. Exposure to TCDD, FICZ, or ITE reduces the number of bone marrow-derived steady-state DCs and the production of IL-6, TNF-*α*, IL-10, and IL-12, while these Ahr agonists do not exert such effects on mature DCs in mice [90]. In the same study, it is shown that TCDD decreases MHCII expression and upregulates CD80, CD86, and CD54, but FICZ and ITE selectively increase the percentage of MHCII, CD86, and CD54 on steady-state DCs. Treating the mice inflammatory bone marrow-derived DCs (BMDCs) with TCDD increases the gene expression of Ahr, TGF-*β*, and IDO [88, 92]. In contrast, inactivated steady-state DCs upregulate gene expression of IDO only [90]. Collectively, it may be suggested that the modulatory effect of Ahr on DCs is population and ligand dependent.

Despite the commonalities they share, DCs and MΦ create discrepancies at certain circumstances. For instance, while lipopolysaccharides (LPS) inhibit the production of IL-10 in MΦ, BMDCs, and splenic DCs from Ahr^−/−^ mice, the CpG inhibits the IL-10 in MΦ [88, 93]. Furthermore, while Ahr expression is induced by LPS and CpG in both BMDCs and peritoneal MΦ, Ahr deficiency enhances IL-6 and TNF-*α* production in the latter only [93]. Mechanistically, the interaction of Ahr with STAT1 negatively regulates NF-*κ*B activation in LPS-stimulated MΦ, which may interpret, at least partly, the enhanced production of the proinflammatory cytokines in Ahr^−/−^ mice [93]. As an alternative pathway independent of STAT1, our data suggest that Ahr negatively regulates the production of IL-6 in MΦ by the suppression of the histamine production [34]. The Ahr/Sp1 heterodimer abrogates Sp1 phosphorylation on Ser residues, which represses histidine decarboxylase expression.

In agreement with the anti-inflammatory role of Ahr, studies using the BMDMΦ and MCF-7 breast cancer cell line show that Ahr mediates the suppression of IL-6 [20, 94], IL-1*β*, and Bcl-2 [94]. In a study conducted using BMDMΦ from LysM-Cre mice, it is suggested that Ahr negatively regulates IL-1*β* production through direct regulation of plasminogen activator inhibitor (Pai)-2 in a NF-*κ*B-dependent mechanism [95].

### 4.3. NK Cells

The NK cells are another diverse population of lymphocytes with emerging functions not only in innate immunity but also in adaptive immunity. The Ahr is expressed in NK cells [96], and the modulatory effects of Ahr in these cells are reported, albeit conflicting results. Treating mice with TCDD exerts no effects on the number of NK cells recovered from the regenerating liver, but it reduces the number of intrahepatic NKT cells [97]. With regard to maturation and function, the authors indicate that TCDD transiently enhances CD69 expression on both NK and NKT cells, but it exerts no effect on the intracellular levels of IFN-*γ* in NK, NKT, or CD3^+^ T cells. In addition, it is found that TCDD augments activation of NKT cell and exacerbates immune-mediated liver injury induced by concanavalin A through a mechanism involving IFN-*γ* and Fas ligand (FasL) [98].

In the absence of Ahr, NK cells show reduced cytolytic activity and capacity to control RMA-S tumor formation *in vivo*, despite having normal developmental and maturation markers [96]. Alternatively, the IDO and Kyn have suppressive effects on the proliferation of NK cells undergoing activation, but not resting cells [99]. Previous work demonstrates that Ahr is indispensable for IDO production [88]. Therefore, it may be postulated that upregulation of Ahr in NK cells has inhibitory effects on cell proliferation via upregulation of IDO.

Among the human NK cell intermediates, stage 3 CD34^−^CD117^+^CD161^+^CD94^−^ cells, so-called immature NK cells, uniquely express Ahr and IL-22 [100]. These cells proliferate in direct response to DC-derived IL-15 and IL-1*β* in secondary lymphoid tissues. It is suggested that IL-1r1^hi^ immature NK cells require exposure to IL-1*β* to sustain the expression of Ahr and IL-22 [100]. Importantly, it is shown in the same study that in the absence of IL-1*β* a greater portion of IL-1r1^hi^ immature NK cells differentiate to stage 4 and acquire the cytotoxic potential and start to secrete IFN-*γ*. Inhibition of Ahr by resveratrol increases the cytotoxic activity of the NK cells by involving activation of JNK and ERK signaling pathways [101]. Taken together, it may be suggested that the inhibitory effects of Ahr on the functions of NK cells may be attributed to arresting immature NK at stage 3. Hopefully, more studies will be conducted to determine the specific condition under which Ahr suppresses the functions of NK cells.

The invariant NKT (iNKT) cells are selected by CD1d and coexpress a restricted TCR repertoire [102]. Other phenotypic features of these iNKT cells include the expression of receptors such as CD161 and NKG2D [103, 104]. Although TCDD treatment does not affect the number of iNKT cells in *Streptococcus pneumoniae*-infected mice [37], recent results using FICZ suggest a modulatory effect of Ahr on cytokines production by human iNKT cells *in vitro* [105]. The iNKT cells have the capacity to produce IL-17. Interestingly, these IL-17-producing cells respond to Ahr activation and express *Il23r* and *RORc* genes [105], similar to conventional Th17 cells. Again, investigating the functions of Ahr in NK cells, *in vivo* and *in vitro*, is still in its infancy. Therefore, more studies may be required to investigate this resemblance between the roles of Ahr in iNKT and Th17 cells and their relevance to autoimmunity.

## 5. Ahr in Barrier Organs

Innate lymphoid cells (ILCs) expressing the nuclear receptor ROR*γ*t are essential source of IL-22 and/or IL-17 that are associated with autoimmunity, reviewed in [106]. The ILCs play vital roles in the defense against intestinal pathogens, in homeostasis of the epithelia, and in the development of intestinal lymphoid follicles and, therefore, protect against inflammatory disorders. Quite recent comprehensive work shows that Ahr is expressed in ROR*γ*t^+^ ILCs and that Ahr signaling is required for the expansion and maintenance of homeostasis of these cells in the gut [107]. The authors also show that mice fed on an indoles-deficient diet are highly susceptible to intestinal pathogen. In agreement, later results show that Ahr-deficient mice succumb to *Citrobacter rodentium *infection and that ROR*γ*t^+^ ILCs from these mice have more apoptosis and less IL-22 production [75]. Therefore, it may be expected that ILCs mediate, at least in part, the modulatory properties of Ahr on autoimmune inflammation.

The receptor tyrosine kinase c-kit is expressed in ROR*γ*t^+^ ILCs, and it is necessary for their cell development and differentiation [108]. Mechanistic investigation reveals that the upstream sequence of the c-kit gene contains functional XREs, which assigns the c-kit a downstream gene of Ahr signaling [23]. An independent study using NKp46^+^ ILCs suggests that the induction of Notch signaling undertakes the Ahr-mediated development of these cells [109]. It remains unclear whether these two mechanisms are results of differential roles of Ahr in these two ILCs subsets.

In addition to the above pointed function in ROR*γ*t^+^ ILCs, the c-kit is required for expansion of the innate *γδ*T cells in the skin [110, 111]. In Ahr-deficient mice, the *γδ*T cells in the skin [111, 112] and intestine [111] do not expand, which is likely attributed to the lower c-kit. Furthermore, it has been suggested that the activation of Ahr by dietary ligands is essential for stable functions of *γδ*T cells in the gut [111]. Taken together, Ahr likely enhances the immunosurveillance of the barrier organs through the c-kit. Additionally, c-kit and its receptor stem cell factor (SCF) are essential to induce proliferation and differentiation of mast cell precursors, reviewed in [113].

Langerhans cells (LCs) are specialized DCs of the epidermis that express higher levels of Ahr and Ahr repressor (Ahrr) [114]. Ablation of Ahr is associated with suppressed IDO and costimulatory molecules CD40, CD80, and CD24a, as well as less granular and smaller LCs [114]. Also, it is shown that Ahr deficiency impairs LCs maturation, which is related to low GM-CSF produced by *γδ*T cells [111]. However, the molecular basis of these observations is lacking.

## 6. Ahr in Inflammation

A large number of inflammatory-related genes have different frequencies of XREs in their promoter regions (Table 1), suggesting modulatory effects of Ahr on the inflammatory responses. Furthermore, several studies have linked inflammation and infection to downregulation of P450 expression using various animal models. As the relation between the expression of P450 and inflammation has been reviewed [30, 115], the current paper addresses the proposed mechanistic details of the regulatory role of Ahr on certain inflammatory mediators.

It is now clear that Ahr regulates inflammatory signals via cross talks with other signaling pathways such as NF-*κ*B pathway. This pathway has long been known to control the expressions of IL-1*β*, IL-6, IL-8, TNF-*α*, and other inflammatory genes. The oxidative stress induced by Ahr ligands may activate the noncanonical NF-*κ*B and AP-1 pathways, resulting in an exacerbated inflammation [116]. Moreover, TCDD augments the production of several chemokines that are known targets of the noncanonical NF-*κ*B pathway such as IL-8, B lymphocyte chemoattractant (BLC), CC-chemokine ligand (CCL) 1, and macrophages chemotactic protein (MCP)-1, which further trigger inflammation [33], and see [30]. Recent study has proposed that TCDD activates the NF-*κ*B pathway by intracellular free calcium in microglial cells resulting in an upregulation of TNF-*α* accompanied by elevation in COX-2 [63].

Alternatively, two recent studies have suggested that Ahr exacerbates inflammation by enhancing the function of the mast cells. One of them suggests that FICZ-exposed human and murine mast cells produce reactive oxygen species, IL-6, and IL-17 in response to cAMP-dependent signals [117]. The other one suggests that Ahr is present in three models of rat mast cell lines and that Kyn enhances the production of IL-6 in RBL2H3 cells [118]. These observations may suggest involvement of mast cells in the modulatory role of Ahr in chronic inflammation and autoimmunity.

On the other hand, more studies have suggested an anti-inflammatory function of Ahr. For example, Ahr-deficient mice show enhanced TNF-*α* production [93]. An upregulation of TNF-*α* may activate the noncanonical NF-*κ*B pathway by a functional interaction between Ahr and RelB [33]. Several *in vivo* and *in vitro* studies, including ours, have documented that Ahr activation reduces IL-12 [59, 90], IL-6 [14, 90], and TNF-*α* [14, 59, 90] in mice and humans [119]. These observations are likely attributed to the inhibitory effect of Ahr/STAT1 on the transcriptional activity of the NF-*κ*B subunit P50 [90] and/or through suppression of histamine production [34]. More recently, we found that activation of Ahr by TCDD *in vitro* and *in vivo* induces cholinergic anti-inflammatory system by upregulating acetylcholinesterase-targeting miR-132 [14]. This system is characterized by decreased proinflammatory molecules including IL-1*β*, IL-6, IL-17, IFN-*γ*, and TNF-*α*.

An earlier report in Nature Medicine suggests mechanisms that may undertake the Ahr-mediated anti-inflammatory effects [120]. The authors suggest that lipoxins attenuate inflammation in infectious diseases in an Ahr/suppressor of cytokine signaling (SOCS)2-dependent manner. In later mechanistic study, they suggest that the lipoxin LXA4 and Kyn trigger an Ahr-dependent SOCS2 expression. In turn, SOCS2 targets TNF receptor associated factor (Traf)-6 by ligation of Lys47 poly-ubiquitin chain and induction of proteasomal degradation, hindering proinflammatory cytokine expression by DCs [121]. Interestingly, both *Socs2* and *Traf6* contain potential XREs boxes in the promoter regions [62], but the physiological significance remains unclear. Additionally, LXA4 and Kyn suppress several proinflammatory genes in a SOCS2-dependent manner, which, consequently, blocks many inflammatory pathways including TLR/MyD88, TLR/TRIF, IL-1r/MyD88, and CD40/CD154 [121].

## 7. Ahr in Murine Models of Autoimmune Diseases

The majority of autoimmune diseases may be prompted by prolonged inflammation, mainly in the individuals who have inherited sensitivity traits. Also, it is now clearly evident that many compounds alter the development of autoimmune conditions via Ahr. The animal models of autoimmune diseases have contributed substantially to achieve this understanding, which may lead to establishing effective treatment in humans.

**Figure 2:**
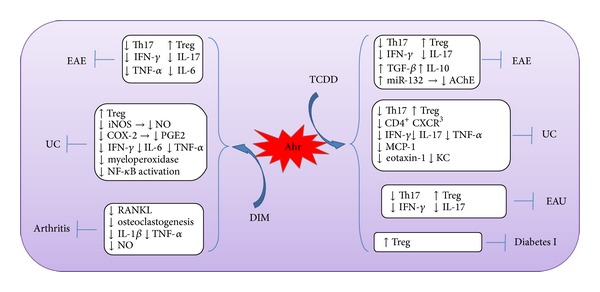
Ahr agonists suppress murine models of autoimmunity. Activation of Ahr by TCDD attenuates experimental autoimmune encephalomyelitis (EAE), ulcerative colitis (UC), experimental autoimmune uveoretinitis (EAU), and diabetes I by promoting differentiation of regulatory T cells (Tregs). With the exception of diabetes I, TCDD inhibits T helper (Th)-17, interferon (IFN)-*γ*, and interleukin (IL)-17. In EAE, Ahr signaling results in upregulation of transforming growth factor (TGF)-*β*, IL-10, and acetylcholinesterase (AChE)-targeting microRNA (miR)-132. In addition to that mentioned above, TCDD downregulates tumor necrosis factor (TNF)-*α*, monocyte chemotactic protein (MCP)-1, and keratinocyte chemoattractant (KC). Activation of Ahr by diindolylmethane (DIM) ameliorates EAE and UC by inducing Tregs and inhibiting proinflammatory cytokines including IFN-*γ*, IL-6, and tumor necrosis factor (TNF)-*α*. Also, the DIM decreases inflammation in UC by inhibiting inducible nitric oxide synthase (iNOS) that produces nitric oxide (NO) and suppressing prostaglandin E2 (PGE2) by inhibiting COX-2, as well as inhibition of myeloperoxidase and nuclear factor (NF)-*κ*B activation. DIM treatment inhibits the expression of receptor activator for ligand (RANKL), leading to the blockade of osteoclastogenesis and consequently an alleviation of experimental arthritis. In addition, the DIM reduces IL-1*β*, TNF-*α*, and NO in arthritis model.

A recently formed paradigm has demonstrated that activation of Ahr by TCDD enhances the CD4^+^FoxP3^+^ cell differentiation. Therefore, TCDD treatment* in vivo* suppresses autoimmune inflammation in several murine models including EAE [13, 14], UC [15], experimental autoimmune uveoretinitis (EAU) [122], and type 1 diabetes [123] (Figure 2). In addition to promoting the differentiation of Tregs, TCDD ameliorates EAE, UC, and EAU by suppressing Th17, IL-17, and IFN-*γ*. Along with these observations, we have found that miR-132 mediates the effect of TCDD on the course of EAE by potentiating cholinergic anti-inflammatory system [14]. However, TCDD is a toxin with unfavorable pharmacological properties, which makes it invaluable therapeutically. Therefore, future research should focus on two approaches: firstly, to design or identify nontoxic Ahr ligands that resemble TCDD and, secondly, to give more emphasis on the identification of the molecular mechanisms that undertake the action of TCDD.

Several groups have demonstrated that natural Ahr ligands attenuate autoimmune inflammation. For example, the dietary indole derivatives indole-3-carbinol (I3C) and DIM promote the expansion of Tregs, while suppressing the induction of Th17 cells in EAE mice [55]. Comparable results are also obtained by DIM treatment in oxazolone-induced colitis [124]. The DIM also alleviates the inflammatory symptoms in a Treg-independent fashion. In DSS-induced colitis, the DIM reduces the disruption of the colonic architecture by suppressing colonic myeloperoxidase activity and the production of proinflammatory cytokines [125]. Furthermore, DIM attenuates experimental arthritis by the inhibition of the receptor activator for nuclear factor *κ*B ligand (RANKL), which leads to blockade of osteoclastogenesis [126].

The endogenous Ahr ligand, ITE, attenuates EAE symptoms by promoting Tregs expansion and inducing tolerogenic DCs that are capable of promoting the Tregs differentiation [89]. Similar results are observed when EAE mice are treated with nanoparticles carrying ITE and MOG_35–55_ [127]. Conversely, treating mice with FICZ or indoxyl 3-sulfate (I3S) worsens EAE, which is likely attributed to the prompted Th17 differentiation [13, 128]. Interestingly, while systematic injection of FICZ does not affect EAE development, local application of FICZ enhances the development of Th17 cells and exacerbates autoimmune conditions [44]. In contrast, mice injected with the FICZ were protected from development of UC and showed decreased proinflammatory cytokines and an increased IL-22 production by Th17 cells [16]. Taken together, it is of importance to assess route of administration of Ahr ligand and to investigate the impact of an individual Ahr ligand in the different autoimmune disease models. Finally, we are confronted with pertinent questions that are toned to be answered. For example, which and under what conditions do the Ahr ligands play a role in attenuation of autoimmune disorders? How can that be applied pharmacologically?

As highlighted earlier in this review, Ahr is critically involved in the differentiation of Th17 and Tregs. Since these cells are reciprocally related, it may be suggested that Ahr is necessary to maintain the balance between these cells under normal conditions, and the augmentation or amelioration of autoimmunity by Ahr ligands is multifactorial including the rout of administration, model used, and the immunological conditions of the host.

## 8. Concluding Remark

It is clear that Ahr is not simply a transcription factor responding to toxins, but it is also critical in the physiological functions of immune cell compartments, in particular Th17, Tregs, and DCs that have prominent roles in inflammation and autoimmunity. A wide range of ligands ranging from small chemicals to dietary derived compounds can modulate the pathogenesis of autoimmune diseases through Ahr signaling. As it is discussed in this review, the epidemiological and mechanistic studies show much discrepancies about the Ahr-mediated regulation of inflammation and autoimmunity. Yet, studying Ahr signaling and alternative pathways is still a valuable approach for future clinical practice.

## Figures and Tables

**Box 1:**
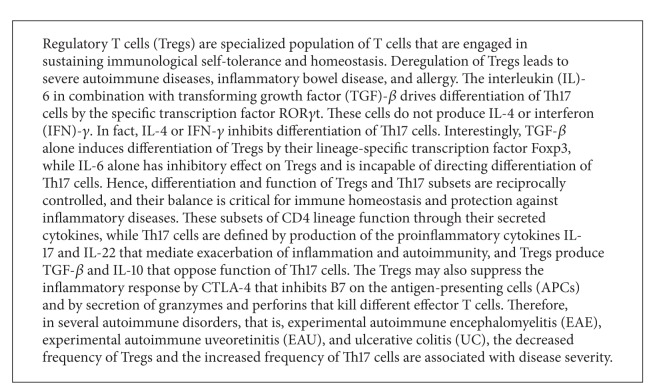
T helper 17 and regulatory T cells.

**Table 1:** Frequency of potential XREs boxes in the upstream sequences of selected inflammatory response-related genes.

Innate immunity		Adaptive immunity	
Gene	XREs frequency	Gene	XREs frequency
*Il6 *	3	*Il2 *	3
*Il18 *	10	*Il4 *	2
*TNF*α**	1	*Il12a *	3
		*Il17 *	3
*Il1r1 *	5	*Il17c *	6
*Il1r2 *	7	*Il21 *	4
*Il1rap *	12	*Il23a *	5
*Il18rap *	1		
		*Il2rb *	7
*Tlr1 *	5	*Il2rg *	5
*Tlr2 *	2	*Il4ra *	9
*Tlr3 *	3	*Il12rb1 *	4
*Tlr4 *	5	*Il17r *	6
*Tlr5 *	9	*Il17rb *	3
*Tlr6 *	3	*Il17rd *	7
*Tlr7 *	4	*Il17re *	6
*Tlr8 *	2	*Ifngr *	11
*Tlr9 *	3	*Ifngr2 *	5
			
*Irak1 *	5	*Jak1 *	5
*Irak4 *	4	*Jak2 *	9
*Traf6 *	5	*Jak3 *	20
*C3ar *	5	*Stat1 *	9
*Il6st *	2	*Stat3 *	5
*Kit *	11	*Stat5a *	9
*Il18bp *	1	*Stat5b *	7
*Lifat *	7	*Irf1 *	6

The genes are selected from [62].
